# Antioxidant and Immune Stimulating Effects of *Allium hookeri* Extracts in the RAW 264.7 Cells and Immune-Depressed C57BL/6 Mice

**DOI:** 10.3390/antiox11101927

**Published:** 2022-09-28

**Authors:** Un-Yul Jeong, Jieun Jung, Eun-Byeol Lee, Ji-Hye Choi, Ji-Su Kim, Hwan-Hee Jang, Shin-Young Park, Sung-Hyen Lee

**Affiliations:** 1 Functional Food Division, Department of Agro-Food Resources, National Institute of Agricultural Sciences, Rural Development Administration, Wanju-gun 55365, Korea; 2 College of Veterinary Medicine, Jeonbuk National University, Iksan 54596, Korea

**Keywords:** *Allium hookeri*, extract, antioxidant activity, immunity

## Abstract

We investigated the antioxidant and immune-enhancing effects of the extracts from *Allium hookeri* leaves and roots (AHL and AHR) in in vitro and in vivo models. Their antioxidant effects were determined by total phenolic content (TPC), DPPH and ABTS radical scavenging activities, and superoxide dismutase and catalase activities. The immunomodulatory effects were evaluated by nitric oxide (NO) production and cytokine concentrations produced from RAW 264.7, and by serum IgA and IgG levels, cytokine levels, and NK cell activities in the immunosuppressed C57BL/6 mice. AHL and AHR extracts improved antioxidant activities and productions of NO and cytokines without cytotoxicity in the RAW 264.7 cells. AHL and AHR groups showed significantly higher serum IgA and IgG levels, Th1 cytokine concentrations, splenocyte proliferations, and NK cell activities than the NC group which was not treated with AHL or AHR extract. AHR extract showed higher values than AHL extract in the factors evaluated in this study. The results show that they have high antioxidant and immunomodulatory effects and can be used as novel potential therapeutic candidates to treat related diseases and to improve public health.

## 1. Introduction

Antioxidants in food systems can retard lipid peroxidation, inhibit oxidation and extend the induction period. They can protect the human body from free radicals and reactive oxygen species (ROS) and give health-promoting effects by preventing related diseases [1,2]. Oxidative stress contributes to human diseases including cardiovascular diseases, chronic kidney disease, and cancer [3]. Inflammation is an innate immune response that leads to adaptive immune responses and is important in maintaining immune homeostasis [4]. Nitric oxide (NO) regulates innate and adaptive immunity [5]. NO depresses or stimulates the pro-inflammatory cytokine expression [6]. The immune system has a complex network of specialized organs, cells, chemicals, proteins and tissues, which has evolved to protect the host from bacteria, viruses, parasites, and cancer cells [7]. The immune system is particularly sensitive to the balance of oxidants and antioxidants because the immune cell functions, especially rely on ROS generation, which is necessary for the cytotoxic activity to protect the body [8]. 

*Allium hookeri* (AH) belonging to the *Allium* genus has been cultured in Korea, China, Sri Lanka, India, Bhutan, and Myanmar [9]. The plant bears white flowers and grows in the months of June to September. It has rhizome-producing fibrous roots and green and thick linear leaves with distinct midribs [9]. AH leaves and roots have been used in spices for foods, such as kimchi, salads, and yogurt as a vegetable with unique flavor and as medicinal herb [10,11]. *Allium* sp. has high amounts of bioactive components such as organosulfur compounds, flavonoids, saponins, and phytosterols. AH includes cycloalliin, alliin, methiin, and isoalliin from organosulfur compounds [9]. It has been reported that AH leaves and roots have various functional characteristics such as anti-oxidant, anti-diabetic, anti-inflammatory, anti-microbial, and anti-obesity effects [12]. Organosulfur compounds are considered major chemical markers in AH, showing anti-inflammatory and anti-obesity in previous studies [9,10,11]. However, there is only limited information available on the anti-oxidant and immune stimulatory effects of AH leaves [13,14] compared with those of AH roots. 

Therefore, we evaluated bioactive component and antioxidant and immunomodulatory effects of AH leaves and roots using in vitro or in vivo experiments with a mouse model, which is close to human physiology, and compared their functional activities. As a responsible bioactive component, cycloalliin content was assessed using LC/MS. The effects were measured by non-enzymatic and enzymatic antioxidant activities and the production of NO and pro-inflammatory cytokines in in vitro studies. Their secretion of immunoglobulins and cytokines, splenocyte proliferation, and NK cell activity were evaluated in the mice immunosuppressed by the cyclophosphamide (CPA). We have focused on comparing antioxidants and immunomodulatory effects of AH leaves and roots. We hope that they can be used as antioxidant and immunomodulatory supplements and as functional food products that can improve public health by controlling related diseases.

## 2. Materials and Methods

### 2.1. Plant Material and Its Responsible Component

#### 2.1.1. Sample Preparation for the Experiment

AH was cultivated in Sunchang-gun and was authenticated by Sunchang Agricultural Development and Technology Center. AH was divided into leaves and roots (AHL and AHR), and they were freeze-dried. AH leaves and roots were added with 10-fold of 50% ethanol and extracted at room temperature (RT) for 24 h. After repeating 2 extraction steps, the AHL and AHR extracts were filtered through filter paper (Whatman NO. 6, Cytiva, Marlborough, MA, USA) and were concentrated by rotary evaporator (EYELA N-1000, Riakikai Co., Tokyo, Japan) at 50 °C. The extracts were freeze-dried (PVTFD 10R, Ilsin Lab, Yangju, Korea) and then stored at −70 °C until experimental use. AH extracts (RDAAHL01, RDAAHR01) were kept in the Functional Evaluation Lab in the Department of Agro-Food Resources. 

#### 2.1.2. Measuring Cycloalliin Concentration

The responsible component for the activity of AH extracts is cycloalliin [9,11]. To analyse the cycloalliin, the extracts (or C_6_H_11_NO_3_S·HCl·H_2_O as a standard, Fujifilm Wako Pure Chemical Co., Osaka, Japan) were dissolved in methanol at 0.1 g/mL. Agilent 6410 Triple Quad LC/MS (Agilent Technologies Inc., Santa Clara, CA, USA) connected to a MS QQQ mass spectrometer with an electrospray ionization source (Agilent Technologies) was used. Chromatographic separations were performed on a reversed-phase C18 with polar endcapping (150 × 2 mm, Synergi^TM^ 4 μm Hydro-RP 80 Å; Phenomenex, Torrance, CA, USA). The temperature was set at 30 °C for an operation and the flow rate was 0.2 mL/min. Mobile phase A and B consisted of 0.1% formic acid in water and 0.1% formic acid in acetonitrile, respectively. The gradient system was as follows: 0 min, 5% B; 1 min, 5% B; 11 min, 100% B; 12 min, 100% B; 15 min, 5% B; 20 min, 5% B. The setting conditions were as follows: gas temperature, 300 °C; gas flow, 11 L/min; nebulizer, 15 psi; capillary, 4000 V. 

MS QQQ mass spectrometer was operated for electrospray ionization (ESI). Detection of the ions was carried out in the multiple-reaction monitoring mode (MRM), by monitoring the transition pairs of m/z 178 → 73. With the help of a thin-layer chromatography technique and computer-assisted image analysis, we performed the quantitative determination of cycloalliin. The protonated fragments at *m*/*z* of 73 and 178 were observed in the positive ESI-MS/MS spectrum, which are consistent with those of the standard compound. 

### 2.2. Evaluation of Antioxidant Activity

#### 2.2.1. Total Phenolic Content 

Total phenolic content (TPC) analysis was modified from the method of Senhaji et al. [13]. Twenty microliters of samples (50, 100, 250, 500, and 1000 μg/mL) were mixed with 80 μL of distilled water and 40 μL of 100% Folin–Ciocalteu’s phenol reagent (Sigma-Aldrich Co., St. Louis, MO, USA) in a 96-well plate, and it was reacted for 3 min. The reacted solution was added with 60 μL of 10% Na_2_CO_3_ and incubated for 2 h. The absorbance was measured at 725 nm using a microplate reader (Molecular Devices, San Jose, CA, USA). Methanol was added to samples and used as a sample blank, and gallic acid (Sigma-Aldrich Co., St. Louis, MO, USA) was used as a standard material. TPC was expressed as μg gallic acid equivalent (GAE)/g of extract.

#### 2.2.2. DPPH Radical Scavenging Activity

Antioxidant activities of AHL and AHR extracts were analyzed by 2,2-diphenyl-1-picrylhydrazyl (DPPH, Sigma-Aldrich Co., St. Louis, MO, USA). The experiment was conducted following the modification of method applied by Senhaji et al. [13]. AHL and AHR extracts were dissolved in methanol at 10 mg/mL and were diluted to 50, 100, 250, 500, and 1000 μg/mL. One hundred microliters of samples were mixed with DPPH solution in 96-well plate and were reacted at RT for 30 min. The absorbance of samples was measured at 517 nm by a microplate reader (Molecular Devices). Samples were mixed with the methanol used as a sample blank and the methanol was used as a control. DPPH radical scavenging ability was calculated by the following formula.

$$\mathrm{DPPH}\;\mathrm{radical}\;\mathrm{scavenging}\;\mathrm{activity}\;\left(\%\right)=(1-\frac{\mathrm{Absorbance}\;\mathrm{of}\;\mathrm{sample}-\mathrm{Absorbance}\;\mathrm{of}\;\mathrm{sample}\;\mathrm{blank}}{\mathrm{Absorbance}\;\mathrm{of}\;\mathrm{control}})\times 100$$

#### 2.2.3. ATBS Radical Scavenging Activity

The 2,2′-azino-bis (3-ethlbenzothioazoline-6-sulfonic acid) diammonium salt (ABTS, Sigma-Aldrich Co., St. Louis, MO, USA) scavenging activities of AHL and AHR extracts were investigated according to the modified method [13]. The ABTS solution was mixed with 7.4 mM ABTS (Sigma-Aldrich Co., St. Louis, MO, USA) and 2.6 mM potassium persulfate (Sigma-Aldrich Co., St. Louis, MO, USA) in equal quantities and were reacted at RT for 20 h in a dark place. The absorbance of ABTS solution was adjusted to 0.70 ± 0.02 at 760 nm. AHL and AHR extracts were dissolved in a methanol at 10 mg/mL and were prepared at different concentrations (50, 100, 250, 500, and 1000 μg/mL). Fifty microliters of samples were mixed with 200 μL of ABTS solution in 96-well plate and then reacted at RT for 10 min. The absorbance was measured at 760 nm by a microplate reader (Molecular Devices). Sample blank was prepared by mixing the methanol with sample and the methanol was used as a control. ABTS radical scavenging ability was calculated by the following formula.

$$\mathrm{ABTS}\;\mathrm{radical}\;\mathrm{scavenging}\;\mathrm{activity}\;\left(\%\right)=(1-\frac{\mathrm{Absorbance}\;\mathrm{of}\;\mathrm{sample}-\mathrm{Absorbance}\;\mathrm{of}\;\mathrm{sample}\;\mathrm{blank}}{\mathrm{Absorbance}\;\mathrm{of}\;\mathrm{control}})\times 100$$

### 2.3. Cell Experiments for Evaluations of Antioxidant and Immunomodulatory Effects 

#### 2.3.1. Measuring Cell Viability

RAW 264.7 cells used in this study were obtained from the Korean Cell Line Bank (Seoul, Korea). Cells were cultured in Dulbecco’s modified Eagle medium (DMEM, Gibco BRI, New York, NY, USA) containing 10% fetal bovine serum (GenDEPOT, Katy, TX, USA) and 1% penicillin-streptomycin (GenDEPOT). Cell viability was analyzed according to Quanti-Max^TM^ WST-8 Cell Viability Assay kit protocol (BIOMAX, Seoul, Korea). RAW 264.7 cells were added to a 96-well plate and incubated for 6 h. Cells were treated with various concentrations (50, 100, 250, and 500 μg/mL) of AHL and AHR extracts and were incubated at 37 °C in an incubator with 5% CO_2_ for 48 h. After removing 100 μL of supernatant in 96-well plate, the remaining 100 μL of cells were mixed with 10 μL of WST-8 reagent and incubated at 37 °C for 1 h. The absorbance was measured at 450 nm using the microplate reader (Molecular Devices).

$$\mathrm{Cell}\;\mathrm{viability}\;\left(\%\right)=\frac{\mathrm{Absorbance}\;\mathrm{of}\;\mathrm{sample}}{\mathrm{Absorbance}\;\mathrm{of}\;\mathrm{control}}\;\times 100$$

#### 2.3.2. Superoxide Dismutase Activity

Superoxide dismutase (SOD) activity was measured by the superoxide dismutase colorimetric activity kit (Invitrogen Co., Waltham, MA, USA). RAW 264.7 cells were seeded in a 6-well plate at a concentration of 2 × 10^5^ cells/well. After incubation at 37 °C for 4 h, AHL and AHR extracts were treated in the RAW 264.7 cells at different concentrations (100, 250, and 500 μg/mL) and were incubated at the same condition for 48 h. The supernatant of media was collected by centrifugation at 1500 rpm for 10 min at 4 °C. In brief, each 10 μL of supernatant was added to 50 μL of the substrate solution with 25 μL of a xanthine oxidase solution in a new plate and the plate was kept at RT for 20 min. The absorbance was measured at 450 nm using a microplate reader (Molecular Devices). The SOD activity was calculated by the standard curve.

#### 2.3.3. Catalase Activity

Catalase (CAT) activity was evaluated using the catalase colorimetric activity kit (Invitrogen Co.). RAW 264.7 cells were seeded in a 6-well plate at a concentration of 2 × 10^5^ cells/well. After incubation at 37 °C with 5% CO_2_ for 4 h, the cells were treated with AHL and AHR extracts at various concentrations (100, 250, and 500 μg/mL) and were cultured in the incubator for 48 h. Cells were centrifuged at 1500 rpm at 4 °C for 10 min and the supernatant was transferred to a new plate. Briefly, 25 μL of samples were mixed with 25 μL of hydrogen peroxide reagent and were reacted at RT for 30 min. The reaction product was added to 25 μL of substrate solution and HRP solution and was incubated at 25 °C for 15 min. The absorbance was measured at 560 nm using a microplate reader (Molecular Devices). CAT activity was calculated by the standard curve.

#### 2.3.4. Nitric Oxide Concentration

NO was measured using the Griess reagent (Sigma-Aldrich Co., St. Louis, MO, USA). RAW 264.7 cells were dispensed in a 96-well plate at 2 × 10^5^ cells/mL and incubated at 37 °C for 6 h. AHL and AHR extracts of various concentrations (50, 100, 250, and 500 μg/mL) were treated into a 96-well plate and were incubated for 48 h. The supernatant and Griess reagent were mixed in equal quantities and were reacted at 25 °C for 10 min. The absorbance was measured at 540 nm using a microplate reader (Molecular Devices). Media and lipopolysaccharide (LPS, 1 μg/mL) were used as negative and positive controls, respectively. Sodium nitrate was used as the standard curve and NO concentration was calculated using the formula bellow:

$$\mathrm{NO}\;\mathrm{content}\;\left(\mu M\right)=\frac{\mathrm{Absorbance}\;\mathrm{of}\;\mathrm{sample}\;-\;0.0059}{0.0499}$$

#### 2.3.5. Cytokine Concentrations Produced by RAW 264.7 Cells

The immunomodulatory effects of AHL and AHR extracts were analyzed using the tumor necrosis factor-α (TNF-α) and interleukin-6 (IL-6) enzyme-linked immunosorbent assay (ELISA) kits (abcam, London, UK). RAW 264.7 cells were seeded at a concentration of 4 × 10⁵ cells/well in 6 well plates and treated with different concentrations of AHL and AHR extracts. After their incubation at 37 °C with 5% CO_2_ for 24 h, the culture was centrifuged at 2000 rpm for 10 min and the supernatant was transferred into a new plate. Fifty microliters of cell supernatants were mixed with 50 μL of cytokine antibody cocktail in each well of the 96-well plate, and then they were incubated at RT for 1 h. The plates were washed 3 times using a wash buffer and 100 μL of the TMB solution was added to each well. They were reacted at RT for 10 min and then the reactions were stopped by 100 μL of a stop solution. The absorbance was measured at 450 nm using a microplate reader (Molecular Devices). The concentrations of cytokine were calculated using the standard curve of each antibody.

### 2.4. Animal Experiment

#### 2.4.1. Experimental Design

**Figure 1:**
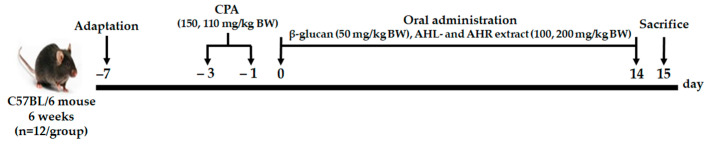
The experimental procedure.

C57BL/6 mice in specific-pathogen-free (SPF) condition were purchased from Central Lab Animal Inc. (Seoul, Korea). They were kept in a controlled environment at 23 ± 2 °C with humidity of 50 ± 10% and 12 h light/dark cycle, and fed normal solid feed and water ad libitum. After one week of acclimation, CPA (Sigma-Aldrich Co., St. Louis, MO, USA) was injected intraperitoneally to induce immunosuppression at 150 and 110 mg/kg before 3 and 1 day of experiment, respectively. The weights of mice were measured after 1 day of 2nd administration with CPA. Mice were randomly selected and divided into 7 groups: (1) normal control group (CON; physiological saline only), (2) negative control group (NC; physiological saline with CPA), (3) positive control group (PC; β-glucan 50 mg/kg BW with CPA), (4) AHL100 group with a low dose of AHL extract (100 mg/kg BW with CPA), (5) AHL200 group with a high dose of AHL extract (200 mg/kg BW with CPA), (6) AHR100 group with a low dose of AHR extract (100 mg/kg BW with CPA), (7) AHR200 group with a high dose of AHR extract (200 mg/kg BW with CPA). The mice were orally administrated with either one of distilled water (DW), β-glucan (Sigma-Aldrich Co., St. Louis, MO, USA), and AH extracts for 14 days (Figure 1). Body and organ weights, serum immunoglobulin and cytokine concentrations, splenocyte proliferation, and NK cell activity of the mice were measured. The animal experiment was conducted under permission and ethical regulations for the animal experiments granted by the Animal Experimentation Ethics Committee (NAS-202106) of the National Institute of Agricultural Sciences.

- Group 1: CON (normal control, distilled water (DW)) (n = 10);
- Group 2: NC (negative control, CPA, DW) (n = 10);
- Group 3: PC (positive control, CPA, β-glucan 50 mg/kg BW) (n = 10);
- Group 4: AHL100 (CPA, AHL extract 100 mg/kg BW) (n = 10);
- Group 5: AHL200 (CPA, AHL extract 200 mg/kg BW) (n = 10);
- Group 6: AHR100 (CPA, AHR extract 100 mg/kg BW) (n = 10);
- Group 7: AHR200 (CPA, AHR extract 200 mg/kg BW) (n = 10).

#### 2.4.2. Collecting Blood and Organs

The body weights of the mice were measured after oral administration with one of DW, β-glucan, and AH extracts for 14 days. Each mouse was anesthetized with CO_2_, and blood was collected from the orbital venous plexus. The blood was centrifuged at 2000 rpm, 4 °C for 15 min, and the serum was used for immunoglobulin and immune-related cytokine analysis. The weights of spleen and thymus were measured and the weights of organs were calculated as the relative weight to the body weight.

#### 2.4.3. Immunoglobulin Concentration in Serum

Immunoglobulin A (IgA) and immunoglobulin G (IgG) concentrations of the mice were analyzed using Mouse IgA and IgG ELISA kits (abcam, London, UK). Fifty microliters of serum and 50 μL of each antibody were added into 96-well plates and were cultured at RT for 2 h. Each plate was washed twice with a washing solution and was added with 50 μL of 1 × HRP antibody. It was incubated at RT for 1 h and then washed. The TMB solution was added to each well at 50 μL and incubated at RT for 15 min. When the color was changed, the reaction was stopped by adding 50 μL of a stop solution. The absorbance was measured at 450 nm using a microplate reader (Molecular Devices). The concentration of each immunoglobulin was calculated by a standard curve.

#### 2.4.4. Cytokine Concentration in Serum

To evaluate the effects of AHL and AHR extracts on blood immune indicators, Th1 cytokines (IL-1β, IFN-γ, TNF-α) and Th2 cytokine (IL-6) were analyzed using ELISA kits (abcam). Fifty microliters of serum and 50 μL of cytokine antibody cocktail were added into a 96-well plate coated with antibody and were incubated at RT for 1h. The plate was washed three times and 100 μL of TMB solution was added to each well. It was reacted for 10 min and the reaction was stopped with 100 μL of a stop solution. The absorbance was measured at 450 nm using a microplate reader (Molecular Devices). Each cytokine concentration was calculated by a standard curve.

#### 2.4.5. Splenocyte Proliferation 

The spleen was washed twice by HBSS (Gibco^TM^, Grand Island, NY, USA) and was homogenized using 40 um nylon cell strainers (BD Biosciences, San Jose, CA, USA). One hundred microliters of spleen lymphocytes were seeded at a concentration of 3 × 10^6^ cells/mL into 96-well plates. Concanavalin A (Con A, 5 μg/mL; Sigma-Aldrich Co., St. Louis, MO, USA) and lipopolysaccharide (LPS, 1 μg/mL; Sigma-Aldrich Co., St. Louis, MO, USA) were added into the wells with splenocytes and were cultured at 37 °C in the incubator with 5% CO_2_ for 48 h. Media was used as a control. Ten microliters of MTS (Promega Co., Madison, WI, USA) were treated in each well and were reacted for 2 h. The absorbance was measured at 490 nm using a microplate reader (Molecular Devices).

#### 2.4.6. NK Cell Activity

Natural killer (NK) cells isolated from the spleen were dispensed into wells of a 96-well plate at 1 × 10^6^ cells/mL, were treated with 1 × 10^4^ cells/mL of Yac-1 cells (effector-to-target 100:1) as target cells, and were incubated at 37 °C in the incubator with 5% CO_2_ for 24 h. NK cell activities were measured by MTS assay kit (Promega Co., Madison, WI, USA). The absorbance was measured at 490 nm using a microplate reader (Molecular Devices). The NK cell activity was evaluated by the cytotoxicity of Yac-1 cells.

$$\mathrm{NK}\;\mathrm{cell}\;\mathrm{activity}\;\left(\%\right)=(1\;-\;\frac{\mathrm{Absorbance}\;(\mathrm{NK}\;\mathrm{cells}+\mathrm{Yac-1}\;\mathrm{cells}\;)\;-\;\mathrm{Absorbance}\;(\mathrm{NK}\;\mathrm{cells})}{\mathrm{Absorbance}\;(\mathrm{Yac-1}\;\mathrm{cells})})\;\times \;100\;$$

### 2.5. Statistical Analysis 

All the samples were carried out in triplicate and analyzed using one-way analysis of variance followed by Duncan’s multiple range test (SPSS ver. 24, IBM Co., Armonk, NY, USA). Data was expressed as mean ± SEM and values were considered as statistically significant at *p* < 0.05.

## 3. Results and Discussion

### 3.1. Concentration of Cycloalliin

**Figure 2:**
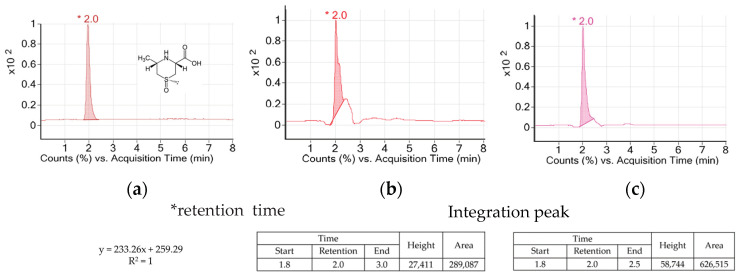
TIC chromatogrmas of cycloalliin in (**a**) standard, (**b**) *A. hookeri* leaves extract and (**c**) *A. hookeri* roots extract analyzed by LC/MS.

AH is a vegetable of the *Allium* genus which is used widely in Asian countries. AH contains special amino acids such as alliin, cycloalliin, and natural compounds including volatile sulfur [14]. Allicin and alkyl thiosulfinate as the sulfur compounds of AH decreased blood glucose and adipogenesis in diabetic models [15,16,17]. Alliin was transformed into thiosulfinates by alliinase during its cooking process such as cutting or crushing. However, cycloalliin was not transformed into thiosulfinate by alliinase and remained in stable condition during the process [18]. So, cycloalliin has been analyzed and used as a chemical and/or biological marker for AH [19]. Figure 2 shows peaks and their heights and areas of the AH extracts. The retention time of cycloalliin was 2.0. The concentrations of cycloalliin (C_6_H_11_NO_3_S) in AHL and AHR extracts were 0.14 and 0.31 mg/g, respectively.

### 3.2. Total Phenolic Content and Antioxidant Activities of AHL and AHR Extracts

Total phenolic content was the major classification of natural antioxidants in plants [20]. TPCs of AHL and AHR extracts were shown in Table 1. At 50~1000 µg/mL of samples, TPCs were ranged from 0.07 to 0.96 µg GAE/g in AHL extract and 0.11 to 1.79 µg GAE/g in AHR extract. TPCs of AHL and AHR extracts increased significantly as an increase in extract concentration and TPC was higher in AHR than in AHL. Previous studies reported that roots had higher TPC than leaves [21,22] and it showed similar pattern compared with our data. However, some studies reported that leaves had higher TPC than roots [23,24,25] and TPC was influenced by the extraction solvent and time [26,27,28]. 

Antioxidant capacity was determined by DPPH and ABTS radical scavenging activities. The DPPH radical scavenging activity is the simplest method to evaluate antioxidant activity of foods and plants [26]. The results of DPPH radical scavenging activity of AHL and AHR extracts are shown in Table 1. The values of AHL extract at 50~1000 µg/mL were 8.58~13.84%, whereas those of AHR extract were 9.23~17.21%. In our previous study, 80% ethanol extracts from AH leaves and roots showed over 50% DPPH radical scavenging activities at 500 μg/mL of samples [13]. DPPH radical scavenging activity was 59.3% at 500 μg/mL of methanolic extract from AH roots [29]. The activity may vary according to the extraction solvent [26,27,28]. ABTS assay measures the relative ability of antioxidants to scavenge the ABTS^+^ generated in aqueous phase. It can be used over a wide range of pH and is a rapid method [30]. The results of ABTS radical scavenging activity assay of AHL and AHR extract are shown in Table 1. Their values of ABTS radical scavenging activities were higher than those of DPPH radical scavenging activities. At 50~1000 µg/mL of extracts, ABTS radical scavenging activities of AHL and AHR ranged from 12.81 to 53.56% and from 11.12 to 83.26%, respectively. Both AHL and AHR extracts showed significant increases in ABTS radical scavenging activities in a dose dependent manner. Many studies have reported that antioxidant effects of vegetables, plants, and herbs evaluated by ABTS radical scavenging activity. Some studies [31,32] showed similar results to our study outcomes of roots extract having higher activity than the leaves extract. The ABTS radical is soluble in aqueous and organic solvents [33]. Therefore, the values may be higher than those of DPPH radical scavenging activity. It was reported that TPC was significantly related to DPPH and ABTS radical scavenging activities. The higher value of TPC was expected to be positively correlated with a higher antioxidant activity [34]. So, DPPH and ABTS radical scavenging activities of AHL and AHR extracts may be improved as an increase in TPC in each extract. 

### 3.3. Enzymatic Antioxidant Effects of AHL and AHR Extracts on RAW 264.7 Cells

**Figure 3:**
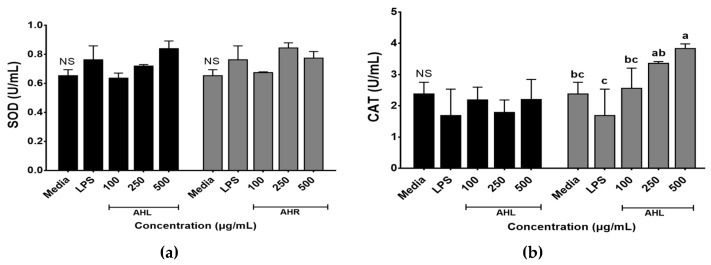
Enzymatic activities of *A. hookeri* leaves (AHL) and *A. hookeri* roots (AHR) extracts in RAW 264.7 cells. (**a**) superoxide dismutase activity (SOD); (**b**) catalase activity (CAT). Results were expressed as the mean ± SEM. ^NS^ Not significantly different. ^a–c^ Different letters on bars are significantly different among CAT values of AHR extract (*p* < 0.05).

Enzymatic antioxidant activities of AHL and AHR extracts were evaluated by SOD and CAT activities (Figure 3). SOD and CAT work to defend cells against oxidative stress [35]. SOD activities ranged from 0.64 to 0.84 U/mL in AHL extract and 0.68 to 0.85 U/mL in AHR extract. No significant difference was found between media and each extract. However, SOD activities of AHL extract at 500 μg/mL and AHR extract at 250 μg/mL increased 1.3 times compared with the media control (Figure 3a). SOD is one of the most powerful antioxidants in the cell and acts as a component of first-line defense system against reactive oxygen species [36]. CAT is a common antioxidant enzyme in all living tissues that utilize oxygen, can break down hydrogen peroxide molecules, and may be enhanced in RAW 264.7 cells by plant extracts [36,37]. Figure 3b showed that the treatment of AHR extract significantly stimulated the CAT production in macrophage cells. CAT activities of AHL and AHR extracts ranged from 1.79 to 2.21 U/mL and from 2.56 to 3.84 U/mL, respectively. CAT activity of AHR extract was higher than that of AHL. There was no significant difference between media and AHL extract. However, CAT activity in AHR extract increased significantly 1.6 times compared with that of the media. Our results indicated that the treatment of AHL and AHR extracts can improve SOD or CAT activity to protect RAW 264.7 cells against oxidative stress. 

### 3.4. Effects of AHL and AHR Extracts on Cell Viability of and NO Production by RAW 264.7 Cells

**Figure 4:**
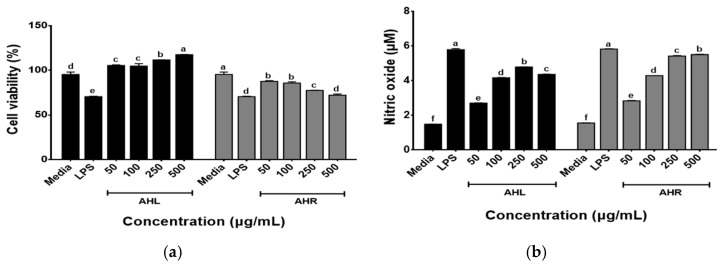
Effects of *A. hookeri* leaves (AHL) and *A. hookeri* roots (AHR) extracts on (**a**) the cell viability of and (**b**) the nitric oxide production by RAW 264.7 cells. The data was expressed as the mean ± SEM. ^a–f^ Different letters on bars are significantly different at *p* < 0.05.

The effects of AHL and AHR extracts on the cell viability of RAW 264.7 macrophages were determined using the WST assay following the treatment of cells with each extract (50, 100, 250, and 500 μg/mL) for 48 h. As shown in Figure 4a, the cell viabilities of AHL extract increased in a dose dependent manner and those of AHR extract recorded over 80% at all concentrations used in this study. The immune-enhancing effects of AHL and AHR extracts were evaluated by the production of NO by RAW 264.7 cells. As shown in Figure 4b, significant changes in NO production after treatment of samples were observed in this study. NO was produced at 2.28~4.80 μM in AHL and 2.85~5.80 μM in AHR. Previous studies have reported that plant extracts including AH extracts stimulated the production of NO in RAW 264.7 or macrophages [13,38,39,40]. NO is a well-known inflammatory mediator and is released from activated macrophages during an infection or inflammation [41]. The extract of AH leaves and roots and fermented AH roots significantly stimulated NO production by HD11 macrophages in a dose dependent manner [13]. NO regulates innate and adaptive immunity by stimulating pro-inflammatory cytokine expression [5,6]. These results suggest that AHL and AHR can stimulate the immune system by NO production in RAW 264.7 macrophages treated with the extracts compared with the media. 

### 3.5. Effects of AHL and AHR Extracts on Cytokine Productions by RAW 264.7 Cells

**Figure 5:**
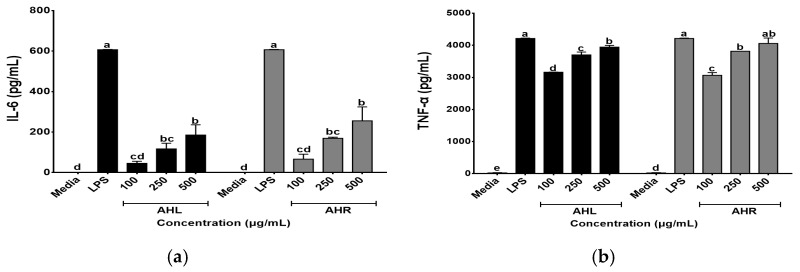
Effects of *A. hookeri* leaves (AHL) and *A. hookeri* roots (AHR) extracts on the productions of (**a**) IL-6 and (**b**) TNF-α by RAW 264.7 cells. The data was expressed as the mean ± SEM. ^a–e^ Different letters are significantly different at *p* < 0.05.

In this study, effects of AHL and AHR extracts on secretions of IL-6 and TNF-α were evaluated using the ELISA kits. As shown in Figure 5, AHL and AHR extracts produced 45.59~185.51 pg/mL and 66.41~255.57 pg/mL of IL-6, respectively. They produced 3164.00 to 3945.64 pg/mL and 3065.00 to 4060.29 pg/mL of TNF-α in AHL and AHR extracts, respectively. The levels of IL-6 and TNF-α were significantly higher than media and increased by AHL and AHR extracts in a dose dependent manner. Macrophages play an important role in the regulation of immune response and this function depends on the secretion of pro-inflammatory cytokines such as IL-6 and TNF-α [42,43]. Previous studies reported that various plant extracts enhanced secretions of the cytokines [44,45]. Thus, AHL and AHR extracts significantly enhanced the productions of IL-6 and TNF-α and can affect immune system by controlling the secretion of pro-inflammatory cytokines. 

### 3.6. Effects of AHL and AHR Extracts on Body and Organ Weights

Table 2 shows the body and organ weights of immunosuppressed mice. The final body weights of the immunodepressed mice were lower than that of the CON mice. However, there was no significant difference found in the values of the immunosuppressed mice treated with or without AHL and AHR extracts. The values of spleen weights were higher in the AHL and AHR groups compared with that of NC group though there was no significant difference in spleen and thymus weights among all groups. Previous study has shown that the administration of aqueous and ethanol AHR extracts increased the spleen weights and NK cell activities, and indicated that they may improve immune system [46]. 

### 3.7. Effects of AHL and AHR Extracts on Immunoglobulin Concentration 

**Figure 6:**
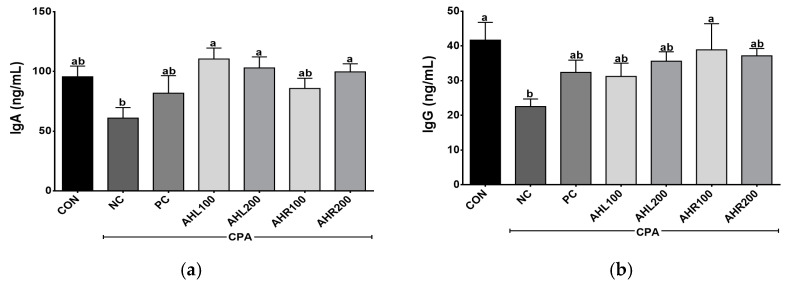
Effects of *A. hookeri* leaves (AHL) and *A. hookeri* roots (AHR) extracts on serum (**a**) IgA and (**b**) IgG levels of C57BL/6 mice immunosuppressed by CPA. CON, normal control group; NC, negative control group; PC, positive control group; AHL100, AHL extract 100 mg/kg BW; AHL200, AHL extract mg/kg BW; AHR100, AHR extract 100 mg/kg BW; AHR200, AHR extract 200 mg/kg BW. Data was presented as the mean ± SEM (n = 12). ^a,b^ Different letters are significantly different at *p* < 0.05.

The serum IgA and IgG levels were shown in Figure 6. AHL and AHR groups showed higher IgA levels than that of the NC group. IgA is the predominant antibody class in the external secretions that bathes mucosal surfaces and plays an important role in immune protection [47]. IgG is an important antibody that recognizes, neutralizes, and eliminates pathogens and toxic antigens and may be enhanced by plant extracts [48,49]. AHR100 group showed the highest IgG level among AH groups and a significant difference was found between NC and AHR100 groups. AH increased the antibodies of inflammatory bowel disease (IBD) and improved immune related microbiome composition, indicating that the AH is a strongly linked to the regulation of immune system [50]. In the present study, the administration of AHL and AHR extracts stimulated the production of IgA and IgG in the immunosuppressed mice, and they may lead to immunomodulatory effects in the mice by improving immune-depressed condition. 

### 3.8. Effects of AHL and AHR Extracts on Cytokine Levels in Serum

**Figure 7:**
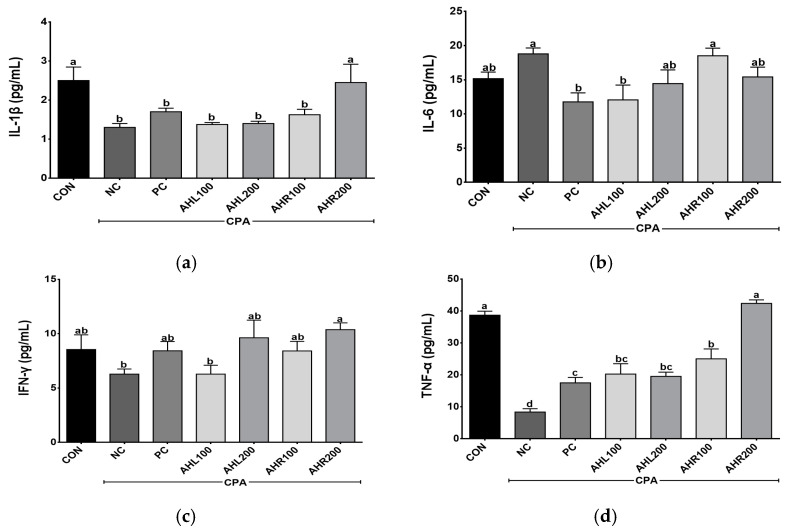
Effects of *A. hookeri* leaves (AHL) and *A. hookeri* roots (AHR) extracts on serum (**a**) IL-1β, (**b**) IL-6, (**c**) IFN-γ, and (**d**) TNF-α levels of C57BL/6 mice immunosuppressed by CPA. CON, normal control group; NC, negative control group; PC, positive control group; AHL100, AHL extract 100 mg/kg BW; AHL200, AHL extract 200 mg/kg BW; AHR100, AHR extract 100 mg/kg BW; AHR200, AHR extract 200 mg/kg BW. Data was presented as the mean ± SEM (n = 12). ^a–d^ Different letters are significantly different at *p* < 0.05.

Th1 (IL-1β, IFN-γ, and TNF-α) and Th2 (IL-6) cytokine levels were determined in the serum of the immune-suppressed mice using ELISA kits. The levels of IL-1β, IL-6, IFN-γ, and TNF-α were shown in Figure 7. The levels of IL-1β, IFN-γ, and TNF-α were higher in the AHR group in a dose dependent manner than in the NC group. The mice treated with AHL and AHR extracts showed similar or higher IL-1β, IFN-γ, and TNF-α levels than those in PC group. IL-6 increased in the NC group compared with the CON group. However, the PC and AHL100 groups showed significantly lower IL-6 levels compared with the NC group and there was a decreasing trend in AHL200 and AHR200 groups. Cytokine binds to a specific cell surface receptor to generate a cell signaling cascade [51]. IL-1β and TNF-α stimulate the production of acute phase proteins and the acute phase of the immune response [50]. IFN-γ has a role in recognizing and eliminating pathogens [52]. IL-6 has unique immune-modifying actions [53]. IL-6 and soluble IL-6 receptor complex induces homodimerization of glycoprotein 130 triggering a downstream signal cascade [54]. IFN-γ and TNF-α levels in the CPA-induced immunosuppressed mice were affected by plant extracts [49]. *A. hookeri* supplementation improved intestinal immune response against necrotic enteritis in young broiler chickens by affecting immune-related cytokines [42]. The serum inflammatory cytokines including IL-1β, IL-6, and IFN-γ, and NF-κB signaling and cytokines in liver tissue were regulated by the administration of AH extracts [55]. *A. hookeri* and *curcuma longa* treatment effectively controlled inflammatory cytokines such as IL-1β, IL-6, and IFN-γ via NF-κB and COX-2 pathways [56]. Th1 cells producing IFN-γ and TNF-α promoted macrophage activation and antibody production [57]. Our study showed similar results to the previous studies that AH extract controlled the production of Th1 and Th2 [50]. Therefore, both leaves and roots extracts from AH can help enhance immunity in the immunosuppressed model by controlling these cytokine levels.

### 3.9. Effects of AHL and AHR Extracts on the Proliferation of Mice Splenocytes

**Figure 8:**
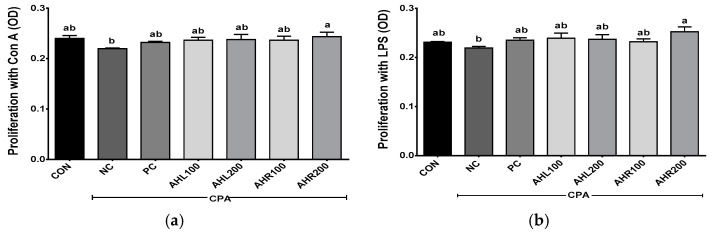
Effects of *A. hookeri* extracts on the proliferation of splenocytes treated with (**a**) Con A and (**b**) LPS in the C57BL/6 mice immunosuppressed by CPA. CON, normal control group; AHL100, *A. hookeri* leaves (AHL) extract 100 mg/kg BW; AHL200, AHL extract 200 mg/kg BW; AHR100, *A. hookeri* roots (AHR) extract 100 mg/kg BW; AHR200, AHR extract 200 mg/kg BW. Data was presented as the mean ± SEM (n = 12). ^a,b^ Different letters are significantly different at *p* < 0.05.

The proliferation was shown in Figure 8. The NC group showed lower levels of splenocyte proliferation after incubations with Con A and LPS, and the administrations of AHL and AHR extracts improved splenocyte proliferations compared with the NC group. AHL and AHR extracts enhanced the proliferation of splenocyte simulated with LPS compared with the NC group. The extracts of AH leaves and roots and fermented AH roots stimulated the proliferation of chicken splenocytes in a dose dependent manner [13]. Spleen is the largest lymphatic organ in the body and serves as an important part of the immune system. It is the site of differentiation and homing of inflammatory macrophages, monocytes, granulocytes, dendritic cells, NK cells, and short-lived plasma cells [58]. The proliferation of splenocytes was improved by the exposure to AHL and AHR extracts. So, it is suggested that AH have immunomodulatory effects and they may be used as materials for immune-enhancing foods.

### 3.10. Effects of AHL and AHR Extracts on the NK Cell Activity in Immunosuppressed Mice

**Figure 9:**
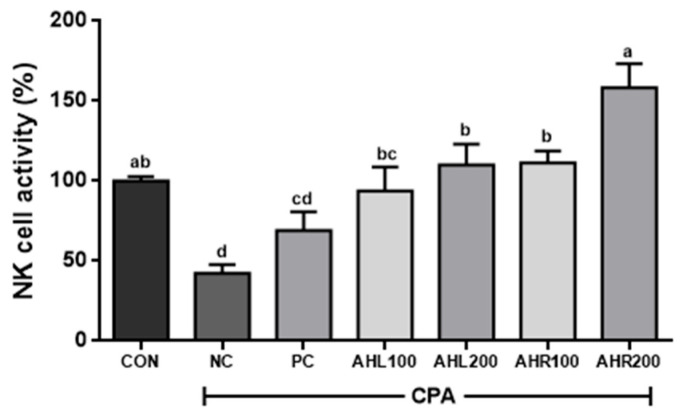
Effects of *A. hookeri* leaves (AHL) and *A. hookeri* roots (AHR) extracts on NK cell activities against Yac-1 in splenocytes of C57BL/6 mice immunosuppressed by CPA. CON, normal control group; NC, negative control group; PC, positive control group; AHL100, AHL extract 100 mg/kg BW; AHL200, AHL extract 200 mg/kg BW; AHR100, AHR extract 100 mg/kg BW; AHR200, AHR extract 200 mg/kg BW. Data was presented as the mean ± SEM (n = 12). ^a–d^ Different letters are significantly different at *p* < 0.05.

As shown in Figure 9, the NC group showed the lowest NK cell activity (42.5%) and AHL and AHR extracts significantly improved NK cell activities compared to the NC group. NK cell activities in AHR100 and AHR200 groups were 111.3 and 158.3%, respectively, and were higher than those (93.8 and 110.1%) of AHL100 and AHL200 groups. AHR200 group showed the highest NK cell activity. The reason that AHR extract showed higher immune stimulating effect rather than AHL extract may be explained by higher amount of cycloalliin in and higher expression of cytokines by AHR.

NK cells are a group of innate immune cells that show cytotoxic activity against tumor cells and virus-infected cells, and secrete INF-γ and TNF-α [59]. The treatment of AH extract increased the NK cell activity in immunosuppressed mice. The cytotoxic activity of NK cells significantly increased after exposure to AH extracts [46]. NK cell activity against Yac-1 was significantly higher in AH groups and it showed similar result to the previous study [60]. It is well known that NK cells can be activated by IFN-γ [53]. In this study, AH extracts may influence NK cell activity by improving the production of IFN-γ in the mice immune-suppressed by CPA and AHR showed relatively higher immune stimulating effects than AHL. Thus, it is suggested that AHR may effectively stimulate immune-system by increasing NK cell activity in the immune-suppressed condition.

## 4. Conclusions

This study has demonstrated that AHL and AHR extracts are good sources of cycloalliin known with high antioxidant and immune modulating effects, and have high total phenolic content, radical scavenging activities, and SOD and CAT activities. They also increased NO production and pro-inflammatory cytokines in RAW 264.7 cells. In in vivo trial, AHL and AHR extracts increased serum IgA and IgG levels, the proliferation of splenocytes, and NK cell activities in CPA-induced immunosuppressed mice. Finally, it is suggested that the ethanolic extracts of AHL and AHR have high antioxidant and immune stimulating effects, and AHR may be effectively used as functional supplements to treat related diseases and to improve public health.

## Figures and Tables

**Table 1:** Total phenolic contents and antioxidant activities of AHL and AHR extracts.

Sample	Concentration (μg/mL)	TPC ^1^ (μg GAE/g)	DPPH Radical Scavenging Activity (%)	ABTS Radical Scavenging activity (%)
AHL extract	50	0.07 ± 0.00 ^e^	8.58 ± 0.37 ^c^	12.81 ± 2.72 ^d^
	100	0.13 ± 0.01 ^d^	8.56 ± 0.30 ^c^	13.32 ± 2.72 ^d^
	250	0.28 ± 0.00 ^c^	9.97 ± 0.59 ^bc^	20.87 ± 3.35 ^c^
	500	0.53 ± 0.01 ^b^	11.60 ± 0.25 ^b^	32.73 ± 1.07 ^b^
	1000	0.96 ± 0.03 ^a^	13.84 ± 0.30 ^a^	53.56 ± 1.28 ^a^
AHR extract	50	0.11 ± 0.00 ^d^	9.23 ± 0.35 ^d^	11.12 ± 0.67 ^e^
	100	0.24 ± 0.04 ^d^	9.37 ± 0.26 ^d^	15.53 ± 0.49 ^d^
	250	0.51 ± 0.01 ^c^	11.00 ± 0.34 ^c^	32.87 ± 0.63 ^c^
	500	0.96 ± 0.04 ^b^	14.01 ± 0.24 ^b^	54.45 ± 0.21 ^b^
	1000	1.79 ± 0.02 ^a^	17.21 ± 0.35 ^a^	83.26 ± 0.52 ^a^

^1^ TPC, total phenolic content. Data was expressed as mean ± SEM. ^a–e^ Different letters are significantly different in each column at *p* < 0.05.

**Table 2:** Effects of *A. hookeri* leaves (AHL) and *A. hookeri* roots (AHR) extracts on the body and organ weights of C57BL/6 mice immunosuppressed by CPA.

	CON ^1^	NC	PC	AHL 100	AHL 200	AHR 100	AHR 200
Initial body weight (g)	23.45 ± 0.24 ^NS^	23.04 ± 0.37	22.87 ± 0.32	22.99 ± 0.20	23.09 ± 0.32	22.97 ± 0.36	23.42 ± 0.39
Final body weight (g)	25.56 ± 0.37 ^a^	24.33 ± 0.31 ^b^	24.05 ± 0.37 ^b^	24.46 ± 0.30 ^b^	24.42 ± 0.41 ^b^	24.11 ± 0.40 ^b^	24.45 ± 0.39 ^b^
Tissue weight (% of BW)							
Spleen	0.26 ± 0.02 ^NS^	0.28 ± 0.01	0.28 ± 0.01	0.30 ± 0.01	0.30 ± 0.02	0.30 ± 0.01	0.30 ± 0.01
Thymus	0.15 ± 0.01 ^NS^	0.17 ± 0.01	0.15 ± 0.01	0.17 ± 0.01	0.17 ± 0.01	0.16 ± 0.01	0.17 ± 0.01

^1^ CON, normal control group; NC, negative control group; PC, positive control group; AHL100, AHL extract 100 mg/kg BW; AHL200, AHL extract 200 mg/kg BW; AHR100, AHR extract 100 mg/kg BW; AHR200, AHR extract 200 mg/kg BW. Data was presented as the mean ± SEM (n = 12). ^NS^ Not significantly different. ^a,b^ Different letters are significantly different among groups at *p* < 0.05.

## Data Availability

Data is contained within the article.
